# LncRNA MALAT1 Targets METTL3 to Orchestrate INPP5B/LRIG2 Signaling and Promote Non‐Small Cell Lung Cancer Progression

**DOI:** 10.1111/cbdd.70421

**Published:** 2026-10-26

**Authors:** Shuhong Tian, Fayu Ling, Dunzhi Fu, Qiongyu Wang, Fan Li, Biao Li

**Affiliations:** ^1^ Hainan Pharmaceutical Research and Development Science Park Hainan Medical University Haikou People's Republic of China; ^2^ Department of Thoracic Surgery The Second Affiliated Hospital of Hainan Medical University Haikou People's Republic of China

**Keywords:** INPP5B/LRIG2 signaling pathway, lncRNA MALAT1, METTL3, NSCLC, targeted binding

## Abstract

m^6^A modification regulates the functions of long non‐coding RNAs (lncRNAs) through diverse mechanisms. However, whether the lncRNA MALAT1 regulates the progression of non‐small cell lung cancer (NSCLC) through targeted regulation of METTL3 remains unclear, and the underlying molecular mechanisms have yet to be elucidated. This study aimed to investigate the role of MALAT1 in NSCLC progression by targeting and regulating METTL3. NCI‐H226 NSCLC cells were used as experimental models. RIP and FISH assays were performed to validate the physical interaction between MALAT1 and METTL3. Cells were co‐transfected with si‐MALAT1 and METTL3 overexpression constructs for rescue experiments. FISH was used to determine the subcellular localization of MALAT1. Apoptotic activity was quantified by flow cytometry, while cell proliferation was assessed using the EdU incorporation assay. The Transwell assay was performed to evaluate cell migration, and the EpiQuik m^6^A RNA Methylation Quantification Kit was used to detect cellular m^6^A methylation levels. Xenograft mouse models were established to evaluate in vivo tumor growth, and clinical NSCLC specimens were analyzed to investigate the clinical correlation among METTL3, INPP5B, and LRIG2. The experimental results showed that MALAT1 was predominantly localized in the nucleus and interacted with METTL3. Following si‐MALAT1 treatment, cell proliferation and migration were decreased, although no significant change was observed in colony formation ability. Furthermore, apoptosis was increased, accompanied by cell cycle arrest at the G0/G1 phase. After MALAT1 interference, transfection with a METTL3 overexpression vector reversed these malignant phenotypes, reduced apoptosis, induced S‐phase accumulation, and significantly downregulated the tumor suppressor gene INPP5B while upregulating the oncoprotein LRIG2. Consistently, in vivo xenograft assays demonstrated that MALAT1 knockdown suppressed tumor growth, which was rescued by METTL3 overexpression. Clinical sample analysis further supported the correlation among METTL3, INPP5B, and LRIG2 in NSCLC tissues. These findings substantiate the regulatory role of MALAT1‐mediated METTL3 modulation in NSCLC progression through the INPP5B/LRIG2 signaling axis.

## Graphical abstract

The long non‐coding RNA MALAT1 acts as a catalyst for non‐small cell lung cancer (NSCLC) progression by targeting METTL3, an interaction that precipitates the downregulation of the tumor suppressor INPP5B.

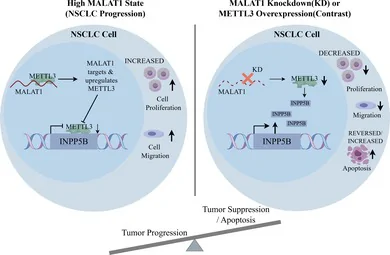

## 1 Introduction

Statistical data indicate that approximately 2.2 million new lung cancer cases are diagnosed annually, resulting in 1.8 million lung cancer‐related deaths worldwide (Deng et al. 2024; Xie et al. 2025; Zhang et al. 2024; Xiong et al. 2025). Non‐small cell lung cancer (NSCLC) represents approximately 85% of all lung cancer cases (Xiong et al. 2025; Wang, Yamada, et al. 2020; Xu et al. 2025). In China, lung cancer is regarded as one of the most malignant tumors, exhibiting high incidence and mortality rates (Tang et al. 2025). Annually, around 828,000 new cases are diagnosed in China, constituting 24.6% of all new cancer cases, with approximately 657,000 deaths, accounting for 29.71% of all cancer deaths (Sung et al. 2021; Castellano et al. 2020). Consequently, NSCLC poses a significant burden on society, families, and individuals.

m^6^A RNA methylation is the most prevalent internal modification found in mRNAs, long non‐coding RNAs (lncRNAs), and microRNAs (miRNAs) within eukaryotic cells (Wilson et al. 2020; Yang et al. 2018; Fan et al. 2026). This modification is introduced by methyltransferases (writers), removed by demethylases (erasers), and recognized by binding proteins (readers) (Ji et al. 2020; An et al. 2024). The m^6^A modification plays a critical role in tumor initiation, proliferation, and metastasis (Liu et al. 2020; Wang, He, et al. 2020; Li et al. 2024).

LncRNAs operate primarily through two mechanisms: transcriptional regulation and post‐transcriptional regulation (Cao et al. 2026). Previous studies have demonstrated that m^6^A modifications frequently occur in lncRNAs (Zhang et al. 2020). While much research has centered on the effects of m^6^A modification‐related proteins on mRNA stability and translation, emerging evidence suggests that certain lncRNAs are capable of modulating the function of m^6^A‐associated methyltransferases in human cancers. However, the regulation of METTL3 and its downstream effectors by MALAT1 in NSCLC remains poorly characterized. Research indicates that LNC942, which is highly expressed in breast cancer (BRCA), promotes BRCA progression by enhancing the mRNA stability of the m^6^A methyltransferase METTL14, which leads to increased METTL14 mRNA and protein expression, subsequently upregulating the expression of downstream target genes such as CXCR4 and CYP1B1 (Sun et al. 2020).

m^6^A modification influences the function of lncRNAs through various regulatory mechanisms (Han et al. 2020; Jia et al. 2019). Specifically, it can act on DNA:RNA triple helix structures, thereby regulating the interaction between lncRNAs and distinct DNA sites. Additionally, m^6^A modification provides binding sites for proteins that recognize methylation (readers) and can regulate the local structure of RNA, facilitating the binding of RNA‐binding proteins (RBPs) and modulating lncRNA functions. Research indicates that the m^6^A methyltransferase METTL3 regulates YAP expression and activity via the lncRNA MALAT1 (LncMALAT1), elucidating a molecular mechanism that contributes to chemoresistance in NSCLC. METTL3 promotes YAP protein translation directly, thereby enhancing YAP protein expression, and also stabilizes MALAT1 by increasing its m^6^A modification levels. Furthermore, MALAT1 acts as a competing endogenous RNA (ceRNA) by binding to miR‐1914‐3p, which subsequently regulates YAP protein expression (Jin et al. 2020, 2021). However, the role of lncRNA MALAT1 in influencing NSCLC progression through targeted regulation of METTL3 remains to be fully elucidated.

In this study, MALAT1 was confirmed to localize in the nucleus and interact with the METTL3 protein. Knockdown of MALAT1 expression resulted in significant decreases in cell proliferation, migration, and colony formation abilities, while apoptosis increased, and G0/G1 phase arrest occurred. Additionally, METTL3 expression was downregulated, along with the oncoprotein LRIG2, while the tumor suppressor protein INPP5B was upregulated, and m^6^A levels decreased. Overall, lncRNA MALAT1 targets METTL3, thereby regulating its expression and subsequently influencing the expression of INPP5B and LRIG2, which affects NSCLC progression.

Jin et al. reported a well‐characterized signaling axis in NSCLC wherein METTL3 deposits m^6^A marks to stabilize MALAT1, which then functions as a ceRNA to sponge miR‐1914‐3p and upregulate YAP, thereby promoting metastasis and drug resistance. In that model, METTL3 is positioned upstream of MALAT1. Conversely, the present study posits that MALAT1 may serve as an upstream regulator of METTL3, functioning through INPP5B/LRIG2 signaling rather than YAP. The potential for MALAT1 to interact bidirectionally with METTL3 and activate alternative downstream signaling pathways in NSCLC remains unresolved, motivating the current mechanistic investigation.

## 2 Materials and Methods

### 2.1 Bioinformatics Analysis

Clinical data for NSCLC patients were obtained from The Cancer Genome Atlas (TCGA) database (https://portal.gdc.cancer.gov/). Original R software was employed for analyses related to m^6^A RNA methylation, tumor classification, PCA, survival, and Cox regression. The ENCORI website facilitated predictions of the targeted binding between MALAT1 and METTL3.

### 2.2 Cell Culture

NCI‐H226 cells (BNCC100405; BeNa Bio) were cultured in RPMI‐1640 medium (KGM31800S; KeyGEN BioTECH) in a 5% CO_2_ incubator at 37°C. Cells reached confluence at approximately 70%, indicating readiness for transfection. The culture medium was replaced with serum‐free RPMI‐1640 (1 mL). Subsequently, 125 μL of Opti‐MEM was added to two sterilized Eppendorf (EP) tubes: 5 μL of Lipofectamine 3000 to one tube and 5 μL of P3000 along with 2.5 μg of the overexpression plasmid to the other. Each tube was mixed and incubated at room temperature for 5 min. The contents of both tubes were then combined and incubated for an additional 15 min before adding the mixture to the corresponding six‐well plates, which were returned to the incubator for culture. Four hours post‐transfection, 1 mL of RPMI‐1640 medium supplemented with 20% serum was added to each well, and subsequent experiments were conducted after 48 h of culture. For survival analysis, patients were stratified into high‐ and low‐expression groups using the median gene‐expression value as a cutoff. Unsupervised consensus clustering was applied for tumor subtype classification. PCA was performed based on the normalized m^6^A regulator expression matrix. Kaplan–Meier survival curves were plotted with accompanying number‐at‐risk tables. Cox proportional‐hazards regression was performed using both dichotomized grouping and continuous gene‐expression values; hazard ratio (HR) and 95% confidence interval (CI) were reported.

### 2.3 Mouse Models

All experimental procedures involving animals received approval from the Animal Ethics Committee of the Research Center for Drug Safety Evaluation of Hainan, Hainan Medical University (approval no. 2022004DE; approval date: April 1, 2022). Testing methods were reported in accordance with ARRIVE guidelines (Yang et al. 2023). Twenty male huHSC‐NCG humanized mice (6 weeks old, weighing 18–22 g) were obtained from Ji Cui Yao Kang (Jiangsu, China) and housed under specific pathogen‐free (SPF) laboratory conditions (temperature 20°C–26°C, humidity 50%–70%), with free access to water and chow. NCI‐H226 cells (1 × 10^6^ cells) were injected subcutaneously into the axilla of the right forelimb after a 7‐day quarantine period. Tumor volumes were subsequently measured every three days using calipers and calculated using the formula: (length × height × width)/2. On day 26 of the experiment, all mice were euthanized using a small animal anesthesia system (Matrx, 5% isoflurane). Prior to subcutaneous injection, NCI‐H226 cells were stably transfected with si‐MALAT1 (si‐1 sequence) or combined with the METTL3 overexpression plasmid (si‐MALAT1 + OE‐METTL3), alongside corresponding negative control groups (si‐NC, si‐MALAT1 + OE‐NC). Stable cell pools were screened and maintained for subsequent in vivo implantation.

The twenty male huHSC‐NCG humanized mice were randomly allocated into four experimental groups (*n* = 5 per group), with randomization occurring before tumor‐cell injection. Tumor volume measurements were conducted by an investigator to group assignment. Power analysis was not performed prior to the animal experiments; the sample size was determined based on previous similar NSCLC xenograft studies. Only male mice were used in this study to reduce variability associated with estrogen‐related hormonal fluctuations; it is acknowledged that this design does not accommodate evaluation of sex‐dependent tumor phenotypes. The experimental endpoint was predefined as day 26 post‐injection due to animal welfare constraints, and survival and distant metastasis outcomes were not assessed in this investigation.

### 2.4 Human Samples

NSCLC and adjacent noncancerous tissues were obtained from patients who underwent surgery at the Second Affiliated Hospital of Hainan Medical University (Haikou, China). This study received approval from the Ethics Committee of Hainan Medical University (approval no. KY105; approval date: April 15, 2022). All study methods adhered to the principles of the Declaration of Helsinki, and informed consent was obtained from each patient prior to sample collection. During surgery, excised tissue samples were rapidly placed in liquid nitrogen.

### 2.5 Immunofluorescence Assay

Cells were fixed with 4% paraformaldehyde and washed three times in PBS within Petri dishes. Subsequently, 5% bovine serum albumin (BSA) was added dropwise to each dish, followed by incubation at 37°C for 30 min. A sufficient volume of diluted primary antibody, specifically the anti‐METTL3 (DF12020, Affinity; 1:200) antibody, was added to each Petri dish, and the dishes were stored at 4°C overnight. Following this, a diluted Cy3‐conjugated fluorescent secondary antibody (Goat Anti‐Rabbit IgG H+L, S007, ABclonal; 1:100) was added, and the dishes were incubated at 37°C for 30 min before thorough washing with PBS. Afterward, 4′,6‐Diamidino‐2‐phenylindole (DAPI) was added, and dishes were incubated in the dark for 3 min for nuclear staining. Observations of the culture dishes were made using a fluorescence microscope (CKX53, Olympus), and images were acquired.

### 2.6 Cell Migration Assay

Cultured cells were resuspended in serum‐free medium and seeded into the upper compartment of a transwell chamber, with medium containing FBS added to the lower compartment. After 24 h of incubation in a CO_2_ incubator at 37°C, the plates were removed, the medium discarded, and the migrated cells stained with 0.1% crystal violet for 1 h. Cells remaining in the chamber were wiped with a cotton swab and observed under a microscope. Following imaging, the staining solution was removed, 33% acetic acid was added, and the absorbance of each well was measured at a wavelength of 562 nm using a microplate reader.

### 2.7 Flow Cytometric Analyses of the Cell Cycle and Apoptosis

Cells (1 × 10^6^) were collected, washed twice with PBS, and centrifuged for 3 min at 1500 rpm. The cells were resuspended in 300 μL of precooled 1× Annexin V‐FITC binding solution, followed by the addition of 5 μL of Annexin V‐FITC and 10 μL of propidium iodide (PI) to each well. The plate was mixed gently and incubated at room temperature for 10 min in the dark. Cell cycle and apoptosis were analyzed using flow cytometry.

### 2.8 Evaluation of Cell Proliferation by a 5‐Ethynyl‐2‐Deoxyuridine (EdU) Incorporation Assay

For EdU incorporation, an equal volume of EdU working solution (20 μM) to culture medium was preheated to 37°C and added to each well of a six‐well plate, which was incubated for 18 h. Afterward, the medium was removed, and cells were washed twice with 1 mL of washing solution per well for 3 min each. A click reaction solution (0.5 mL) was then added to each well, and the plate was incubated for 30 min. The click reaction solution was removed by centrifugation, and the cells were washed with the washing solution three times for 3 min each before resuspending in 500 μL of PBS for analysis.

### 2.9 m^6^A RNA Methylation Assay

For the m^6^A RNA methylation assay, total RNA was extracted from the cells, and m^6^A levels were measured using an EpiQuik m^6^A RNA Methylation Quantification Kit (Colorimetric, Base Catalog # P‐9005). A serial dilution standard curve was generated according to the manufacturer's instructions, with total RNA input normalized across all samples. The global m^6^A level was calculated as a percentage of m^6^A relative to total RNA.

### 2.10 Fluorescence in Situ Hybridization (FISH)

Cellular localization of MALAT1 and METTL3 was determined using FISH. Paraffin sections were deparaffinized with xylene and rehydrated, then treated with pepsin diluted in citric acid (3%). In situ hybridization was conducted using MALAT1 (CY3, MK3969‐h) and METTL3 in situ hybridization kits (Boster). Fluorescence staining was performed with a Cy3‐conjugated fluorescent secondary antibody (Boster), and nuclear staining was conducted with DAPI.

### 2.11 RNA Immunoprecipitation (RIP) Assay

Cells were lysed, and lysates were pretreated with magnetic beads for 30 min. An anti‐METTL3 antibody (15073‐1‐AP, Proteintech), a negative control antibody (rabbit IgG, GB111738, Servicebio), and a positive reference antibody (U1 snRNP 70, sc‐390899, Santa Cruz Biotechnology) were added, followed by incubation with magnetic beads for 3 h. An immunoprecipitation (IP) kit (cat. no. P0102, GENESEED) was used for antigen capture for 2 h. The magnetic bead mixture was washed, RNA was purified and extracted, and quantitative PCR (qPCR) samples were analyzed using a thermal cycler. Input RNA samples were reserved prior to immunoprecipitation for qPCR normalization, with U1 snRNP 70 serving as the positive IP control and rabbit IgG as the negative control. IP enrichment efficiency was evaluated via qPCR relative to input.

### 2.12 RNA Isolation and Reverse Transcription–Quantitative PCR (RT–qPCR)

TRIzol reagent was used to extract total RNA from cells, while mRNA/lncRNA was isolated using an RNA ultrapure extraction kit. RNA concentration and purity (OD260/OD280) were assessed via a UV–visible spectrophotometer. cDNA was synthesized with an RNA RT kit, and fluorescence qPCR was conducted using a fluorescence PCR system. The reaction consisted of 40 cycles: denaturation at 95°C for 10 min, followed by denaturation at 95°C for 10 s, annealing at 58°C for 30 s, and extension at 72°C for 30 s. Relative gene expression was calculated using the 2^−△△Ct^ method, with β‐actin serving as the internal reference. The sequences of the primers used for target gene amplification were as follows:

5′ TGGCACCCAGCACAATGAA 3′ (β‐actin F);

5′ CTAAGTCATAGTCCGCCTAGAAGCA 3′ (β‐actin R);

5′ TGGAGTTGGGGAGAGAATGTCTA 3′ (METTL3 F);

5′ TTTCCTTTGACACCAACCAAGC 3′ (METTL3 R);

5′ ACACAGCAAGCAAACAGGTCAT 3′ (INPP5B F);

5′ GCCATCAAGTAGTGGAAGACATTT 3′ (INPP5B R);

5′ GGGAGATACCATGATCACGAAGGT 3′ (RIP Positive primer‐U1‐F);

5′ CCACAAATTATGCAGTCGAGTTTCCC 3′ (RIP Positive primer‐U1‐R);

5′ AAATCCGTGAGGTCGGCAAT 3′ (LncRNA MALAT1 F);

5′ TCTCCAGGACTTGGCAGTCT 3′ (LncRNA MALAT1 R).

### 2.13 Protein Extraction and Western Blot (WB) Analysis

Western blot analysis was conducted to quantify target protein levels, normalized to β‐actin levels. Cell samples were harvested for lysis, placed on ice for 15 min, and centrifuged for 10 min at 12,000 rpm. The supernatant was combined with buffer solution, boiled for 5 min, and stored at −20°C. Protein concentration in the supernatant was determined using a bicinchoninic acid (BCA) quantitative kit. A 10% sodium dodecyl sulfate–polyacrylamide gel electrophoresis (SDS‐PAGE) resolving gel and a 5% stacking gel were prepared, followed by loading protein samples and a marker into the wells for electrophoretic separation. The gel was then cut and positioned in a transfer system for membrane transfer at a constant current of 300 mA for 90 min. Subsequently, the membrane was blocked with 3% skim milk at room temperature for 1 h. The polyvinylidene fluoride (PVDF) membrane was incubated overnight with a mouse anti‐β‐actin antibody (HC201, TransGen Biotech; 1:2000). After washing, the PVDF membrane was incubated with horseradish peroxidase (HRP)‐conjugated goat anti‐rabbit IgG (GB23303, Servicebio; 1:2000) for 2 h. Following another wash, the membrane was immersed in a chemiluminescence solution and placed in the sample area of an ultrasensitive chemiluminescence imaging system to initiate the imaging program.

### 2.14 Statistical Analysis

All in vitro experiments were performed with at least three independent biological replicates, each consisting of multiple internal technical replicates. Quantitative data were analyzed using IBM SPSS Statistics 26.0. Two‐group comparisons were conducted using independent‐sample *t*‐tests, while one‐way ANOVA followed by Tukey post hoc tests was applied for multiple‐testing corrections during multi‐group comparisons. *p* < 0.05 was considered statistically significant. *p < 0.05; **p < 0.01; ***p < 0.001.

## 3 Results

### 3.1 Clinical Significance of the m^6^A RNA Methyltransferase Gene METTL3

Data for NSCLC tumors and adjacent paracancerous tissues were retrieved from the TCGA database for bioinformatics analysis. Expression levels of m^6^A RNA methyltransferase genes in tumor tissues were significantly higher compared to those in paracancerous tissues (Figure 1A). Based on these expression levels, tumor samples were categorized into two groups: group 1 (low expression) and group 2 (high expression) (Figure 1B). Principal component analysis (PCA) was subsequently performed (Figure 1C). Survival analysis based on the expression levels of m^6^A RNA methyltransferase genes indicated that lower expression correlated with longer patient survival times (Figure 1D). Cox regression analysis identified the m^6^A RNA methyltransferase gene METTL3 as having the most significant impact on NSCLC patient prognosis (Figure 1E). A further survival analysis revealed a significant correlation between METTL3 expression levels and patient survival; specifically, lower METTL3 expression was associated with longer survival (Figure 1F).

**FIGURE 1:**
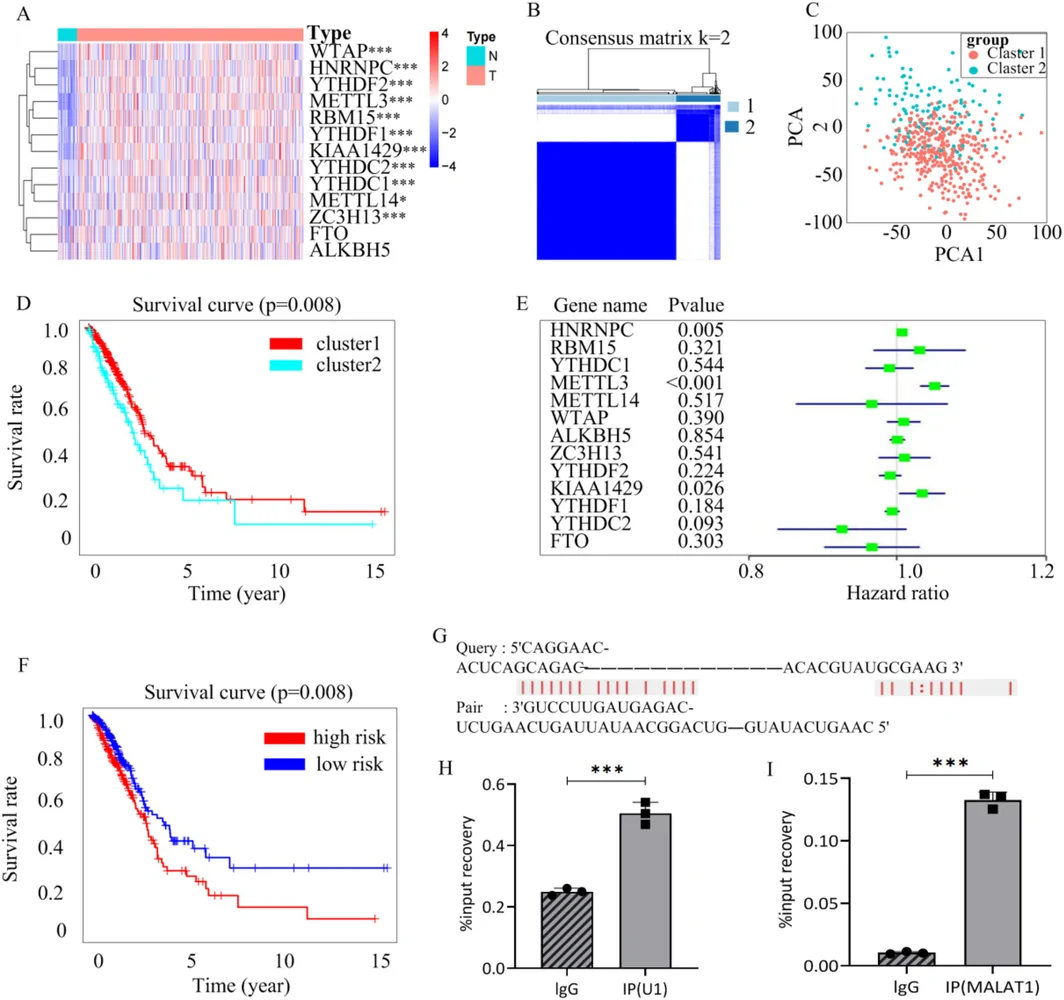
Analysis of m^6^A RNA methylation in clinical data from non‐small cell lung cancer patients. (A) Differential analysis of m^6^A RNA methyltransferase gene expression. (B) Tumor classification based on m^6^A RNA methyltransferase gene expression levels. (C) PCA performed on tumor classification. (D) Survival analysis based on m^6^A RNA methyltransferase gene expression in different tumor subtypes. (E) Cox regression analysis based on m^6^A RNA methyltransferase gene expression. (F) Survival analysis of NSCLC patients according to METTL3 gene expression levels. (G) Prediction of targeted binding of lncRNA MALAT1 to METTL3. (H, I) RIP assays confirming the targeted binding of lncRNA MALAT1 to METTL3. The statistical significance values indicate *p < 0.05, ***p < 0.001.

### 3.2 MALAT1 Interacts With METTL3

Our previous study indicated a close relationship between METTL3 and MALAT1 expression. The potential for targeted binding between MALAT1 and METTL3 was predicted using ENCORI (Figure 1G). The RIP assay confirmed that MALAT1 binds to METTL3 (Figure 1H,I). These findings suggest that METTL3 expression in NSCLC is significantly associated with patient survival and prognosis, possibly linked to the targeted binding of lncRNA MALAT1.

Subsequent analysis involved studying the colocalization of MALAT1 and METTL3 in NSCLC cells, utilizing FISH to assess the effects of MALAT1 interference and METTL3 overexpression. Results demonstrated nuclear colocalization of MALAT1 and METTL3. LncRNA MALAT1 was predominantly expressed in the nucleus, and METTL3 mRNA also localized primarily in the nucleus under low expression conditions. However, following METTL3 overexpression, a portion of the METTL3 mRNA was observed in the cytoplasm (Figure 2).

**FIGURE 2:**
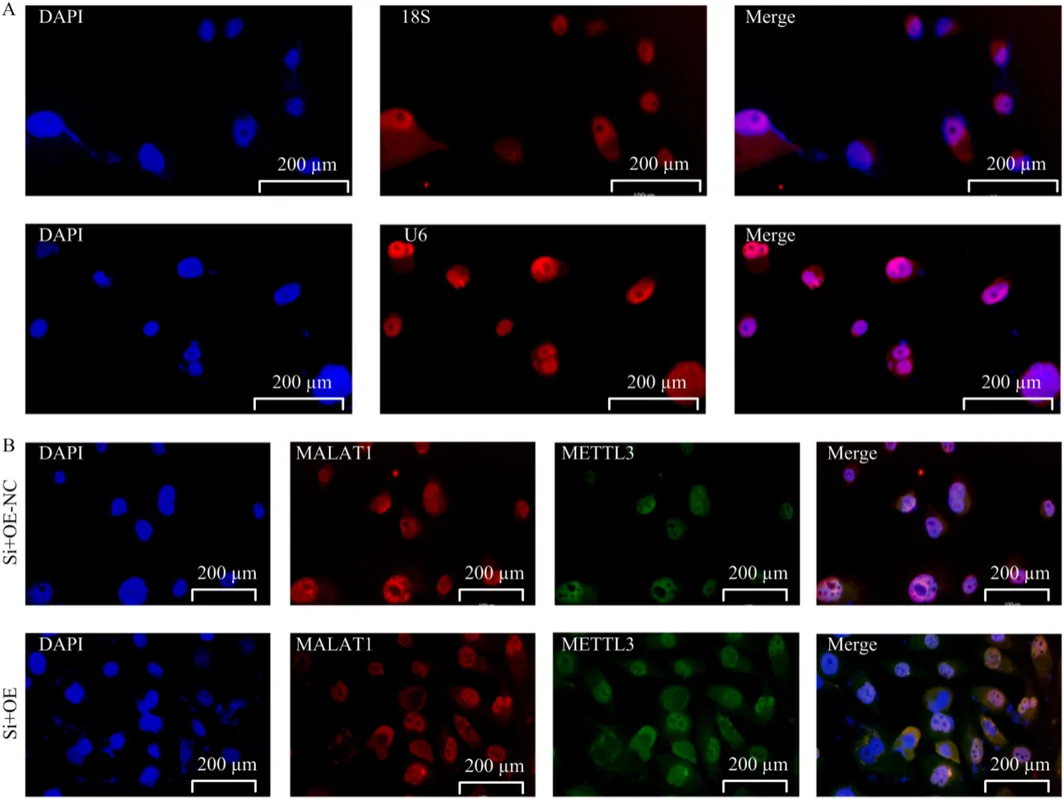
FISH assays confirming the subcellular localization of lncRNA MALAT1 and METTL3 in non‐small cell lung cancer (NSCLC) cells. (A) 18S rRNA served as a cytoplasmic localization reference; U6 snRNA served as a nuclear localization reference; DAPI stains the cell nuclei. (B) Colocalization of MALAT1 and METTL3 in NSCLC cells across two experimental conditions: Si+OE‐NC (si‐MALAT1 plus empty overexpression vector control) and Si+OE (si‐MALAT1 plus METTL3 overexpression plasmid). Si and OE represent si‐MALAT1 and OE‐METTL3, respectively.

### 3.3 Effect of MALAT1 on the Phenotype of NSCLC Cells

To investigate the effect of lncRNA MALAT1 on NSCLC, three targeted interference sequences were designed based on the human lncRNA MALAT1 sequence from GenBank (NR_002819.4, Gene ID: 378938): Si‐1, Si‐889, and Si‐1808. The silencing efficiency of the Si‐1 interference sequence proved to be the highest (Figure 3A). RNA electrophoresis results clearly displayed distinct bands for 18S RNA and 28S RNA, with an intensity ratio of 2:1, indicating good RNA integrity (Figure 3B). Additionally, DNA electrophoresis results confirmed that both β‐actin and MALAT1 bands exhibited expected intensities and correct locations, corresponding to the target products (Figure 3C). Consequently, Si‐1 was selected for the subsequent generation of si‐MALAT1 cells.

**FIGURE 3:**
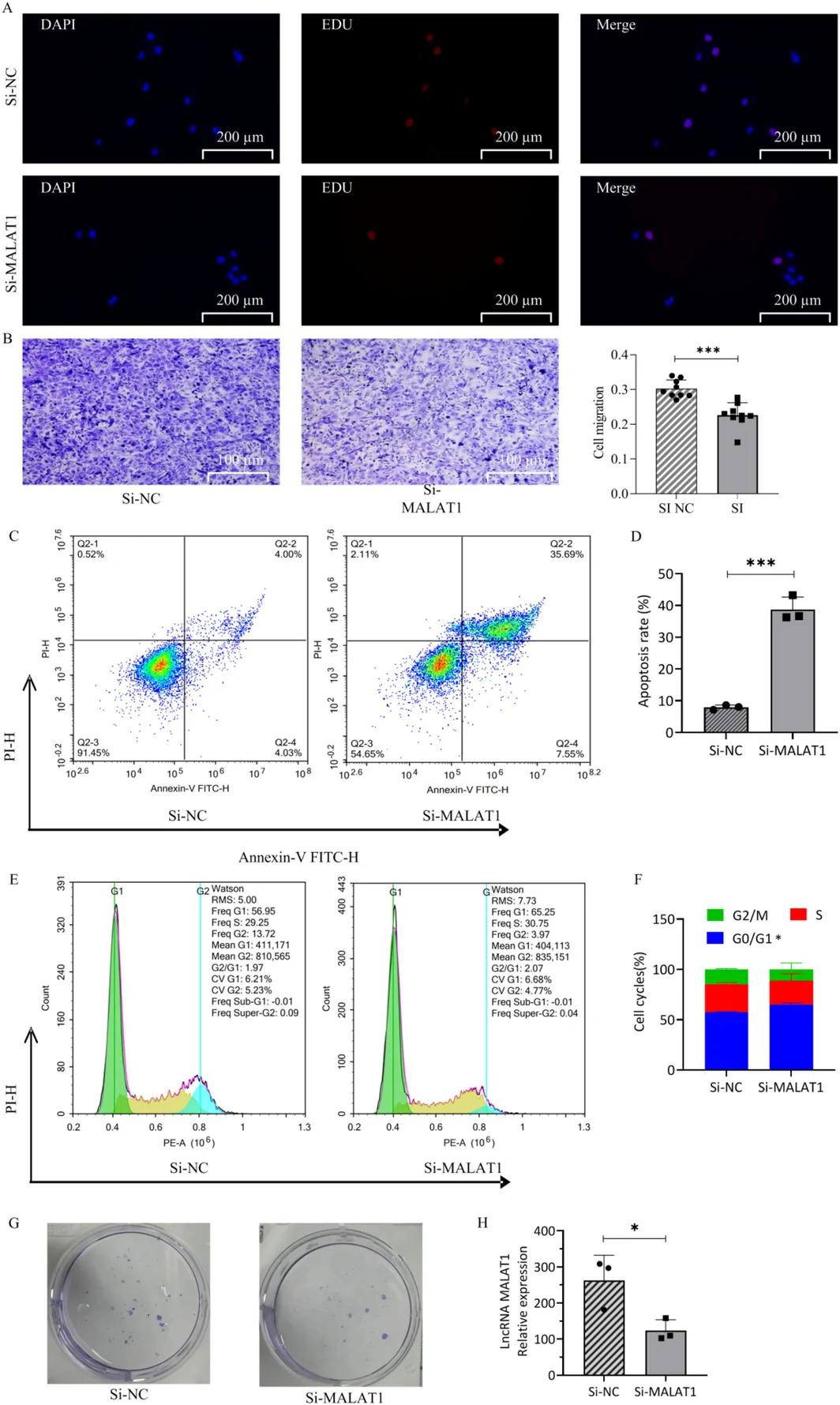
The effect of lncRNA MALAT1 on the phenotype of non‐small cell lung cancer cells. (A) Immunofluorescence analysis assessing the proliferation ability of NSCLC cells transfected with siRNA targeting MALAT1 (*n* = 3). (B) Transwell migration assays evaluating cell migration capability. (C) Cell cycle analysis via flow cytometry. (D) Quantitative analysis of the flow cytometry data presented in C. (E) Flow cytometry utilized for apoptosis analysis. (F) Quantitative analysis of the apoptosis data in F. (G) Colony‐forming ability evaluation through colony formation assays. (H) Quantitative analysis of the colony formation data in G. The statistical significance values indicate *p < 0.05, ***p < 0.001.

Following the silencing of lncRNA MALAT1 in the NSCLC cell line NCI‐H226, various assays were performed to evaluate cell characteristics. Proliferation ability was assessed using an immunofluorescence assay, migration capability was evaluated through a transwell migration assay, and cell cycle distribution as well as apoptosis were analyzed using flow cytometry. Colony formation ability was assessed via a colony formation assay. Post‐silencing of lncRNA MALAT1, a significant decrease in cell proliferation was observed (Figure 3D), alongside a notable reduction in cell migration (Figure 3E), increased apoptosis (Figure 3F,G), G0/G1 cell cycle arrest (Figure 3H,I), and a decrease in colony formation ability (Figure 3G,H). These results suggest that inhibition of lncRNA MALAT1 impedes NSCLC progression.

### 3.4 Effect of MALAT1 on METTL3, INPP5B, and LRIG2 Expression

Next, this study confirmed the effect of lncRNA MALAT1 on the expression of the m^6^A RNA methyltransferase METTL3 in NSCLC cells. Following interference with lncRNA MALAT1 expression, qPCR results indicated a significant decrease in MALAT1 mRNA transcript levels (Figure 4A), while immunofluorescence staining revealed a notable decrease in METTL3 protein expression (Figure 4B). This study further examined the impact of lncRNA MALAT1 interference on the tumor suppressor INPP5B and the tumor‐promoting protein LRIG2 using transcriptome sequencing. Western blot analysis demonstrated that silencing MALAT1 resulted in a significant upregulation of INPP5B protein expression and a downregulation of LRIG2 protein expression (Figure 4C–E). Additionally, interference with MALAT1 expression led to a significant decrease in cellular m^6^A levels (Figure 4F). These findings suggest that MALAT1 regulates the expression of the tumor suppressor gene INPP5B and the oncoprotein LRIG2 in NSCLC cells, thereby influencing the overall m^6^A RNA methylation levels in the cells.

**FIGURE 4:**
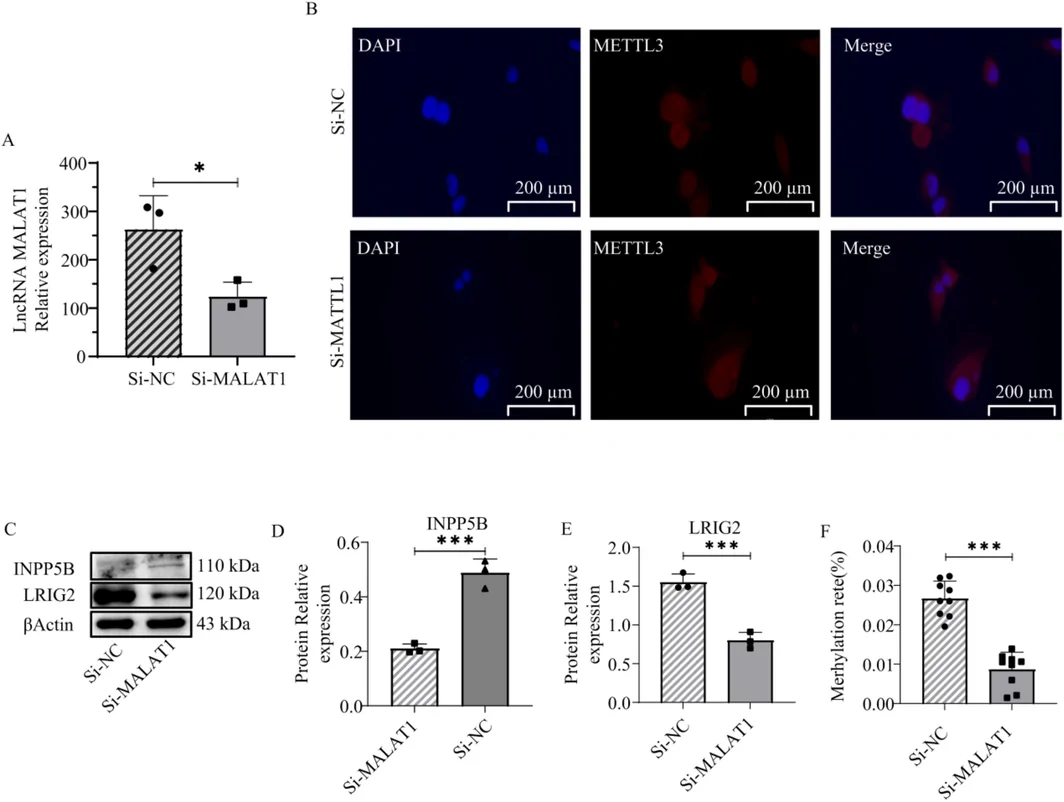
Effect of lncRNA MALAT1 on the expression of METTL3, INPP5B, and LRIG2. (A) qPCR analysis of lncRNA MALAT1 expression in NSCLC cells transfected with siRNA targeting MALAT1 (*n* = 3). (B) Immunofluorescence analysis assessing METTL3 protein expression. (C) Western blot analysis of INPP5B and LRIG2 expression in NSCLC cells following MALAT1 silencing (*n* = 3). (D) Quantitative analysis of INPP5B expression from data in C. (E) Quantitative analysis of LRIG2 expression in data from D. (F) Quantitative analysis of m^6^A RNA modification in NSCLC cells with MALAT1 silencing (*n* = 9). The statistical significance values indicate *p < 0.05, ***p < 0.001.

To further validate that lncRNA MALAT1 may affect NSCLC progression by targeting the m^6^A RNA methyltransferase gene METTL3, a METTL3 overexpression plasmid (Figure 5A) was constructed and transfected into NSCLC cells with MALAT1 silencing. Subsequent measurements of mRNA and protein expression levels for METTL3, INPP5B, and LRIG2 were performed. The mRNA expression of METTL3 was significantly upregulated in the cells (Figure 5B), and Western blot results corroborated that METTL3 protein expression was also significantly increased (Figure 5C). Furthermore, the expression of INPP5B protein was significantly downregulated (Figure 5D), while the expression of LRIG2 protein was significantly upregulated (Figure 5E). These results imply that lncRNA MALAT1 regulates INPP5B and LRIG2 expression in NSCLC cells through targeted binding to METTL3, reinforcing the role of the m^6^A RNA methyltransferase gene METTL3 in promoting NSCLC progression.

**FIGURE 5:**
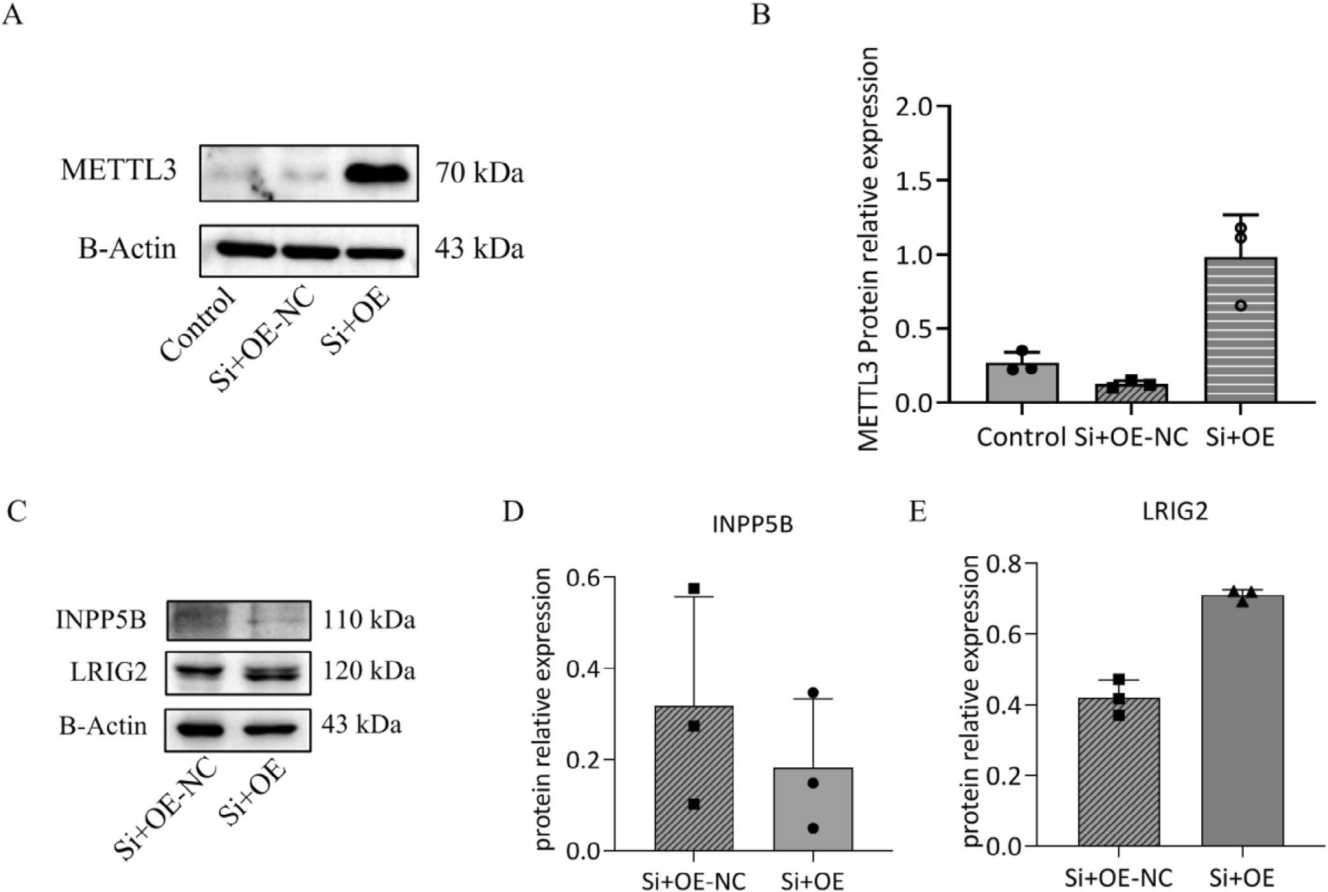
Effect of METTL3 overexpression on non‐small cell lung cancer cells with MALAT1 silencing. (A) Western blot analysis of METTL3 expression (*n* = 3). (B) Quantitative analysis of data from A. (C) Western blot analysis of INPP5B and LRIG2 expression (*n* = 3). (D) Quantitative analysis of INPP5B expression from data in C. (E) Quantitative analysis of LRIG2 expression from data in C. Si and OE represent si‐MALAT1 and OE‐METTL3, respectively.

Next, this study assessed the effects of METTL3 overexpression on the proliferation, migration, cell cycle, and apoptosis of NSCLC cells. Results showed a significant increase in cell proliferation capacity (Figure 6A), which paralleled an increase in cell migration capability (Figure 6B,C). Apoptosis was significantly reduced (Figure 6D,E), and cells exhibited S‐phase arrest (Figure 6F,G). Furthermore, while interference with MALAT1 expression inhibited NSCLC tumor growth, METTL3 overexpression promoted tumor growth (Figure 6H,I).

**FIGURE 6:**
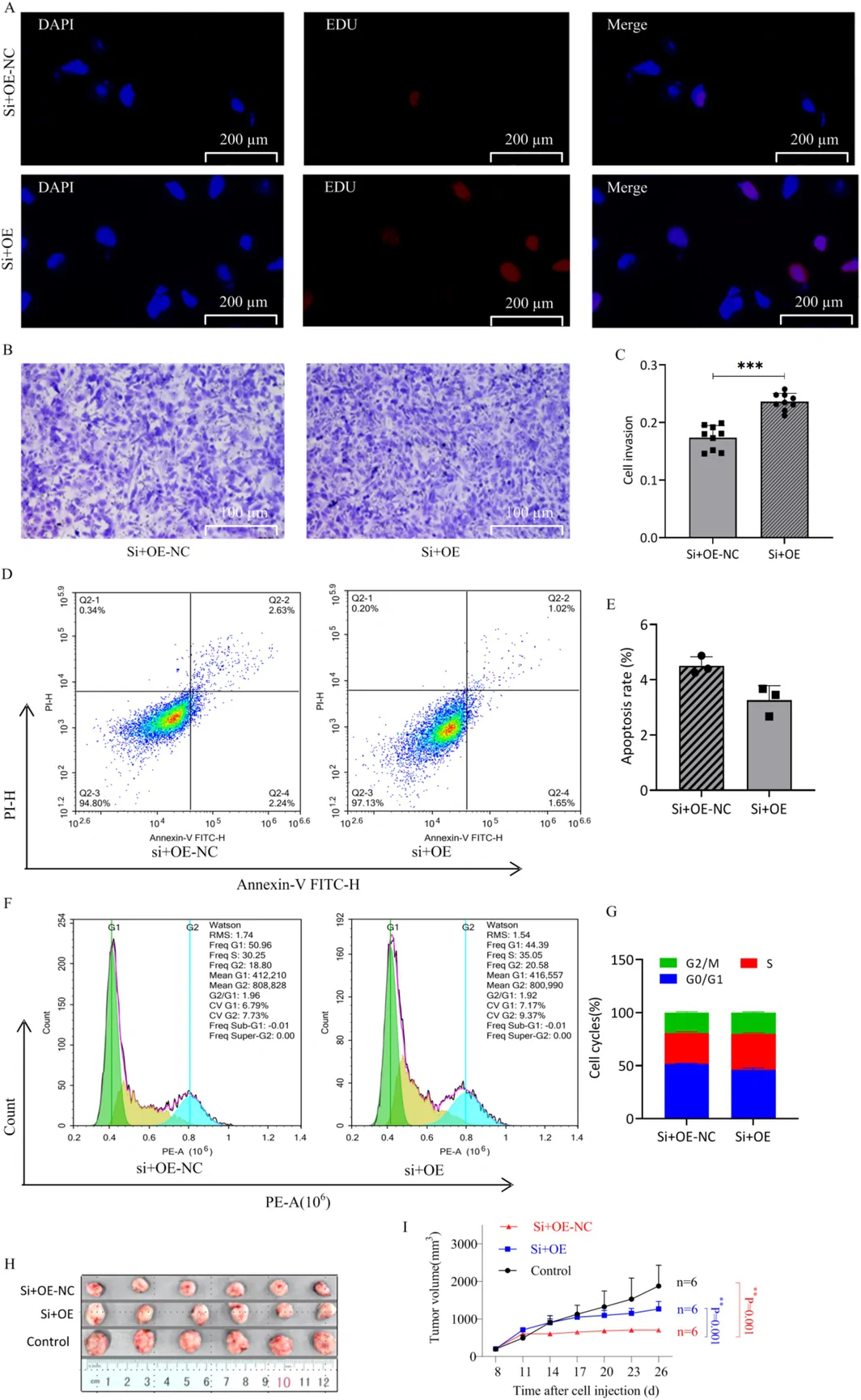
Effect of METTL3 overexpression on the phenotype of non‐small cell lung cancer cells with MALAT1 silencing. (A) Effect on cell proliferation. (B) Effect on migration ability. (C) Quantitative analysis of data in B. (D) Effect on apoptosis. (E) Quantitative analysis of data in D. (F) Effect on the cell cycle. (G) Quantitative analysis of data in F. (H) Photographs of tumors. (I) Growth curve of tumors. Si and OE represent si‐MALAT1 and OE‐METTL3, respectively. ***p < 0.001.

### 3.5 Correlation Analysis of INPP5B, LRIG2, and METTL3 Expression in Clinical Samples

Previous in vitro experiments investigated the regulatory effects of METTL3 on NSCLC and the underlying mechanisms. Subsequently, the cellular localization of METTL3 and its correlation with the expression levels of INPP5B and LRIG2 in clinical NSCLC samples were analyzed. FISH results demonstrated that METTL3 localized to the nucleus (Figure 7A–C). Immunohistochemical analysis revealed that INPP5B protein expression was downregulated while LRIG2 protein expression was upregulated in clinical samples exhibiting high METTL3 expression (Figure 7D–K). These findings further support that the m^6^A RNA methyltransferase gene METTL3 facilitates NSCLC progression by inhibiting tumor suppressor INPP5B expression and enhancing the expression of the oncoprotein LRIG2.

**FIGURE 7:**
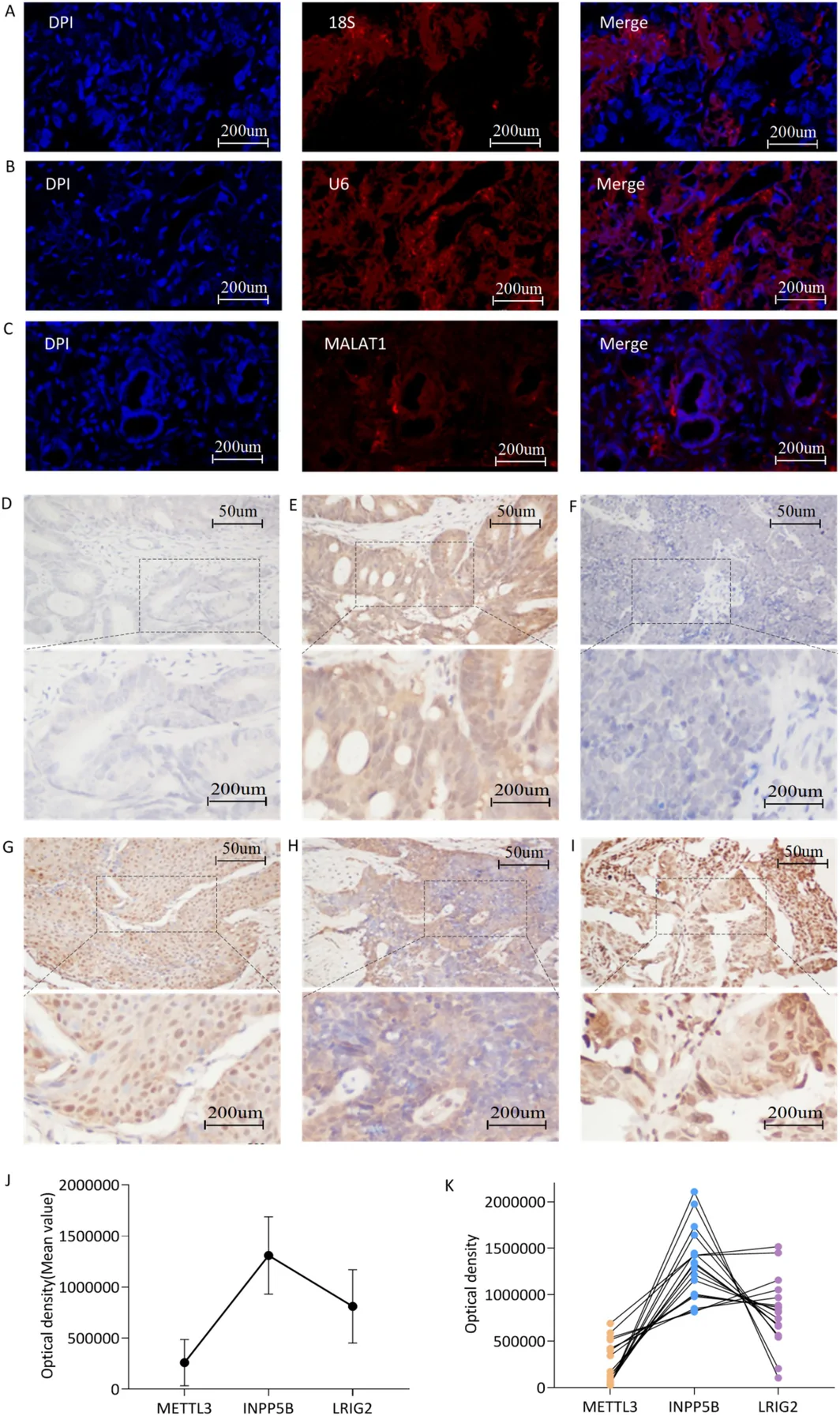
Cellular localization of METTL3 in clinical NSCLC samples and correlation analysis of METTL3 expression with INPP5B and LRIG2 expression. (A) Cellular localization of 18S, a cytoplasmic marker. (B) Cellular localization of U6, a nuclear marker. (C) Cellular localization of METTL3. (D) Immunohistochemical staining indicating low METTL3 expression. (E) Immunohistochemical staining showing high INPP5B expression. (F) Immunohistochemical staining indicating low LRIG2 expression. (G) Immunohistochemical staining displaying high METTL3 expression. (H) Immunohistochemical staining showing low INPP5B expression. (I) Immunohistochemical staining indicating high LRIG2 expression. (J) Quantitative analysis of immunohistochemical data (means and standard deviations). (K) Quantitative analysis of immunohistochemical data (all data).

## 4 Discussion

Previous studies in colorectal cancer, osteosarcoma (OS), multiple myeloma, and NSCLC have outlined a canonical regulatory cascade in which METTL3 functions as an upstream m^6^A writer, installing m^6^A modifications on MALAT1 to enhance its transcript stability. However, current findings indicate a reverse regulatory direction in NCI‐H226 NSCLC cells, where lncRNA MALAT1 acts upstream to target and upregulate METTL3, thereby governing the INPP5B/LRIG2 signaling axis. This discrepancy may be partially attributed to tumor‐type or cell‐context specificity. Distinct cellular backgrounds could drive divergent molecular interactions between MALAT1 and METTL3, resulting in different downstream effector pathways. Unlike earlier studies that focused on METTL3‐dependent stabilization of MALAT1, the present study identifies MALAT1 as an upstream positive regulator of METTL3 and characterizes INPPB/LRIG2 as downstream functional effectors in NSCLC, constituting a major novelty of this work. The possibility of bidirectional crosstalk between MALAT1 and METTL3 cannot be excluded; further investigation is necessary to dissect the temporal–spatial dynamics of this regulatory loop.

Comparison with prior work by Jin et al. highlights a canonical METTL3‐MALAT1‐miR‐1914‐3p‐YAP signaling axis in NSCLC (Jin et al. 2021). In their model, METTL3 is upstream of MALAT1, stabilizing its transcript through m^6^A modification to drive drug resistance and metastasis (Jin et al. 2021). In contrast, the current study reveals a reverse regulatory direction, wherein MALAT1 acts upstream to positively regulate METTL3, operating through INPP5B/LRIG2 rather than YAP signaling. These two models are not mutually exclusive. A bidirectional regulatory loop between MALAT1 and METTL3 may exist, with cell status, tumor subtype, or spatial–temporal context influencing the predominant regulatory direction. To date, limited reports have characterized m^6^A modification sites on INPP5B or LRIG2 transcripts (Hu et al. 2022; Deng, Lin, et al. 2022). Whether other m^6^A methyltransferases can modulate INPP5B/LRIG2 expression warrants further exploration.

Both in vitro and clinical data indicate that METTL3 modulation is correlated with altered protein abundance of INPPB and LRIG2 (Hu et al. 2022; Deng, Lin, et al. 2022). However, direct evidence of m^6^A methylation on INPP5B or LRIG2 mRNA is currently lacking. It remains unproven whether METTL3 regulates INPPB/LRIG2 through direct m^6^A modification or via indirect intermediate signaling molecules. Future investigations utilizing m^6^A‐RIP‐qPCR combined with site‐directed mutagenesis will be essential to clarify these mechanistic details.

The m^6^A modification plays an active role in maintaining tumor cell function and characteristics during tumorigenesis and development (Liu et al. 2022; Cheng et al. 2023). METTL3, as a core component of the m^6^A methyltransferase complex, exhibits methyltransferase activity and serves as a catalytic subunit associated with mitogen‐activated protein kinase cascades, ubiquitin‐dependent degradation, RNA splicing, and various cellular regulatory processes (Guo et al. 2022; Tang et al. 2023). High METTL3 expression correlates positively with poor tumor differentiation, advanced tumor stage, and metastasis. Notably, METTL3 expression is significantly elevated in NSCLC tissues compared to normal paracancerous tissues (Zhang et al. 2023, 2021; Jiang et al. 2022). Furthermore, studies indicate that knockout of endogenous METTL3 alters the abundance and profile of m^6^A in mRNAs, which directly affects colony formation of H1299 cells (an NSCLC cell line) in soft agar and the growth of xenograft tumors (Yang et al. 2023).

The mechanisms of action of lncRNAs encompass both transcriptional and post‐transcriptional regulation. Research has demonstrated that m^6^A modification is prevalent in lncRNAs within tumor cells (Deng, Deng, et al. 2022; Li et al. 2022). Previous studies on NSCLC have centered primarily on the effects of the m^6^A methyltransferase METTL3 on mRNA stability and translation (Feng et al. 2023; Zhang et al. 2022; Wu et al. 2022). However, limited research has explored the involvement of lncRNAs in the regulation of METTL3 and their impact on METTL3 target gene expression and the tumor immune microenvironment during tumor development, thereby influencing tumor progression. This study reveals that MALAT1 targets METTL3, regulating its expression and affecting NSCLC progression. Silencing MALAT1 expression led to a downregulation of METTL3 and an inhibition of NSCLC cell growth.

LRIG2 is highly expressed in OS tissues and cell lines, with downregulation of LRIG2 significantly inhibiting the proliferation and migration of OS cells while increasing apoptosis (Hu et al. 2022). INPP5B, conversely, is downregulated in various solid tumors, particularly lung adenocarcinoma (LUAD). Its overexpression significantly inhibits the proliferation and migration of LUAD cells and blocks G2/M transition (Deng, Lin, et al. 2022). Our results indicate that after MALAT1 expression interference, METTL3 expression was downregulated, resulting in a significant decrease in the expression of the oncoprotein LRIG2 and a significant upregulation of the tumor suppressor protein INPP5B. Following METTL3 overexpression, LRIG2 expression significantly increased, while INPP5B expression significantly decreased, a finding corroborated by NSCLC clinical samples. These results suggest that the expression of LRIG2 and INPP5B may be regulated via METTL3‐mediated methylation.

In conclusion, findings from this study affirm the regulatory influence of METTL3 on the oncoprotein LRIG2 and the tumor suppressor protein INPP5B. However, the precise mechanisms underlying this regulation warrant further investigation. Future research will focus on determining whether m^6^A modification serves as a direct mechanism linking METTL3 to its downstream targets.

## 5 Limitations

This study has several limitations. First, all cellular experiments were conducted exclusively using the NCI‐H226 cell line, necessitating validation in additional NSCLC cell lines to confirm the generalizability of the observations. Second, the clinical sample cohort is relatively small; despite supplementing with clinicopathological parameters and performing multivariate adjustments, larger independent cohorts are needed to validate the clinical correlation between METTL3, INPP5B, and LRIG2 (Hu et al. 2022; Deng, Lin, et al. 2022). Third, xenograft assays were exclusively performed in male mice, limiting the evaluation of sex‐dependent tumor phenotypes. Fourth, while the current RIP assay demonstrates a physical interaction between MALAT1 and METTL3, the absence of RNA pull‐down/RNA‐EMSA and domain‐truncation mutagenesis prevents formal confirmation of direct binding. Fifth, catalytic‐dead METTL3 mutant constructs were not utilized; thus, it is impossible to definitively differentiate the protein‐level function of METTL3 from its methyltransferase catalytic activity. Additionally, reciprocal rescue experiments and single‐gene manipulation of INPPB/LRIG2 were not conducted. Evidence of direct m^6^A modification on INPPB and LRIG2 mRNA is also lacking. Although findings suggest the MALAT1‐METTL3 axis as a potential therapeutic target for NSCLC, in vivo targeting of lncRNA‐methyltransferase interactions encounters several practical challenges, including delivery efficiency, off‐target effects, and concerns regarding drug stability. Concrete therapeutic intervention strategies will require extensive follow‐up pre‐clinical investigations.

## 6 Conclusion

In summary, this study demonstrates that lncRNA MALAT1 binds to and positively regulates METTL3 in NSCLC cells, which in turn modulates the tumor suppressor INPP5B and oncogenic LRIG2, driving NSCLC malignant phenotypes both in vitro and in xenograft models. The findings reveal a reverse regulatory direction compared to the previously reported METTL3‐driven MALAT1‐stabilization axis in NSCLC, highlighting the context‐dependent interaction between MALAT1 and METTL3. Correlations from clinical samples further substantiate the clinical relevance of the METTL3‐INPP5B‐LRIG2 signature. However, the direct m^6^A‐mediated regulation of INPPB/LRIG2 transcripts by METTL3 requires further experimental validation. These findings provide new mechanistic insights into understanding the lncRNA‐m^6^A methyltransferase regulatory network in NSCLC.

## Author Contributions

**Shuhong Tian:** writing – original draft, conceptualization, formal analysis, visualization, supervision, data curation. **Fayu Ling:** conceptualization, methodology, writing – original draft, validation. **Dunzhi Fu:** methodology, investigation, software, formal analysis. **Qiongyu Wang:** investigation, visualization, software, supervision. **Fan Li:** writing – original draft, formal analysis, data curation, validation, methodology. **Biao Li:** funding acquisition, validation, conceptualization, writing – review and editing, project administration, resources.

## Funding

This work was supported by Hainan Provincial Natural Science Foundation High level Talent Project (Grant 822RC843).

## Disclosure

Preprint declaration: A preprint version of this article is available at Research Square: https://doi.org/10.21203/rs.3.rs‐5243760/v1. This preprint has not been peer‐reviewed.

## Ethics Statement

This study was approved by the Ethics Committee of the Hainan Medical University (approval no. KY105; approval date: April 15, 2022). All experimental procedures involving animals were approved by the Animal Ethics Committee of the Research Center for Drug Safety Evaluation of Hainan, Hainan Medical University (approval no. 2022004DE; approval date: April 1, 2022). This study was conducted in accordance with the Declaration of Helsinki (as revised in 2013).

## Conflicts of Interest

The authors declare no conflicts of interest.

## Data Availability

The raw data can be obtained on request from the corresponding author.
